# Reconciliation of Work and Personal Roles Among Critical Care Nurses: Constructivist Grounded Theory Research

**DOI:** 10.3390/healthcare13101206

**Published:** 2025-05-21

**Authors:** Miguel Valencia-Contrera, Lissette Avilés, Naldy Febré

**Affiliations:** 1 Faculty of Nursing, Andrés Bello University, Santiago 8370146, Chile; naldy.febre@unab.cl; 2 Nursing Studies Department, University of Edinburgh, Edinburgh EH8 9AG, UK; lissette.aviles@ed.ac.uk

**Keywords:** work–life balance, nursing, intensive care units, qualitative research, grounded theory

## Abstract

Objectives: There are numerous and varied theoretical gaps in the study of work–family interaction, which limits the understanding and approach to this phenomenon, especially among vulnerable groups such as critical care nursing professionals. In this regard, the objective of this study was to theorize the work–family interaction process among nurses working in Critical Care Units in Chile. Methods: Constructivist grounded theory was employed to conceptualize the phenomenon of interest from the perspectives of nurses, their families, and administrative staff. Data were collected through observations in two high-complexity hospitals in Chile, one public and one private; document analysis; and 51 in-depth interviews. Data analysis was conducted using constant comparisons and multi-level coding. To ensure rigor, the study followed the 13 criteria proposed by Charmaz and Thornberg for constructivist grounded theory studies and was approved by a Scientific Ethics Committee. Results: The reconciliation of work and personal roles emerged as the core process explaining the interaction between work and personal life among nurses in Critical Care Units. This complex and multidimensional process comprised three stages: resisting the war of roles, hitting rock bottom, and reconciling, through which nurses attempt to achieve balance between work and personal roles. Conclusions: We developed a theory that explains the work–family interaction process. The theory developed highlights the importance of an integrated approach that considers both sources of conflict and reconciliation strategies. Addressing this phenomenon effectively requires interventions at the individual, organizational, and public policy levels.

## 1. Introduction

Workers are exposed daily to various occupational health and safety risks, among which psychosocial risks are particularly significant. These are defined as “interactions between and among work environment, job content, organizational conditions and workers’ capacities, needs, culture, personal extra-job considerations that may, through perceptions and experience, influence health, work performance and job satisfaction” [1].

Among psychosocial risks, the work–family conflict stands out as the only one that explicitly considers workers’ personal and family dimensions, yet it remains one of the least explored areas [2]. Theoretical models in this field first emerged at the end of the 20th century [3], and since then, research has primarily focused on three conceptual approaches [4]: I. work–family conflict, which considers work and family as incompatible and antagonistic domains; II. work–family balance, referring to strategies or practices aimed at harmonizing or balancing the demands of both domains; and III. work–family interaction, which acknowledges the bidirectional influence between family and work spheres, with both positive and negative potential outcomes.

The International Labour Organization has recognized the significance of work–family interaction, introducing Convention No. 156 in 1981 concerning workers with family responsibilities. This aimed to support employees across all sectors of economic activity [5]. Chile ratified the convention on October 14, 1994, and it remains in force to this day [6].

In the Chilean context, national legislation mandates the use of the Copenhagen Psychosocial Questionnaire to assess psychosocial risks at work, with the Superintendency of Social Security responsible for publishing the results. According to the most recent report from 2024, based on 2023 data, the highest percentage of workplaces classified as high-risk are found in the health sector, at 8.0%. This situation becomes more concerning when considering the combined percentages of medium- and high-risk categories (suboptimal risk), where health sector workplaces again rank highest, reaching 45.4% [7]. Specifically, in the work–life balance dimension, the health sector shows the highest proportion of workers exposed to suboptimal risk, at 89.7%.

Within the health sector, nursing professionals have been particularly affected, reporting higher levels of work–family conflict compared to other occupational groups [8]. This can be partially attributed to the profession’s predominantly female composition [9], accounting for approximately 90% of the global workforce [10], combined with hostile working conditions that hinder family caregiving roles [11].

In the Chilean context, cross-sectional studies have characterized hospital nurses as having an average age of 32 years and less than 7 years of professional experience [12]. Furthermore, regarding nurses’ working conditions, it has been reported that these do not always align with the recommendations of the Ministry of Health, for example, with respect to adequate staffing levels in Intensive Care Units (ICUs) [13].

Nurses working in ICUs are more affected than those in general wards, mainly due to the demanding nature of critical care work. This environment is characterized by high physical demands, continuous stress, and emotional exhaustion, which often hinders the ability to disconnect from work and fully engage in family life [14,15].

Despite the relevance of this phenomenon, several gaps in its study limit an adequate response, highlighting the need for theoretical proposals that contribute to the exploration of psychosocial risks [16,17,18,19]. In particular, within the work–family interaction domain, there is a need to deepen the understanding of the organizational factors involved [20]. Furthermore, a recent literature review has identified both theoretical and empirical gaps in the field, emphasizing the lack of an integrated vision of work and non-work domains [21]. On one hand, the work domain has primarily been addressed through dichotomous perspectives, while non-work domains have been largely reduced to caregiving and family responsibilities.

Addressing these theoretical gaps is crucial to managing occupational psychosocial risks. However, the limited development of this area is understandable, given the inherently complex nature of the phenomenon. The study of family, in itself, constitutes a complex unit of research, as does the study of working life; thus, analyzing their interaction implies a synergistic level of complexity [22]. This study aims to theorize the work–family interaction process among nursing professionals working in ICUs in Chile, employing a constructivist grounded theory approach. This study forms part of a doctoral dissertation and, as such, presents a partial report of the findings.

## 2. Materials and Methods

### 2.1. Study Design

A constructivist grounded theory was used [23]. This constructivist grounded theory is an inductive approach that incorporates strategies from the original grounded theory [24], while incorporating distinctive elements [25]: 1. it assumes a relativist epistemology; 2. it recognizes that both the researcher and research participants possess multiple perspectives, roles, and realities; 3. it adopts a reflexive stance toward the researcher’s background, values, actions, situations, relationships with participants, and representations of them; and 4. it situates the research within the historical, social, and situational conditions of its production.

Data collection was conducted by the principal investigator (MVC) in 2024 as part of their doctoral training in Nursing Science. The researcher has prior experience in the field of occupational health and safety, particularly in psychosocial risk factors, which enhanced their theoretical sensitivity to the research phenomenon.

Grounded in symbolic interactionism as a theoretical and methodological framework, this study assumes that society, reality, and the self are constructed through interaction and are therefore dependent on language and communication [26]. The study is reported using the COREQ guideline (Supplementary File S1). For further details regarding the methodological decisions of the study, it is recommended to consult the research protocol [27].

### 2.2. Researcher Positioning

As previously discussed, by adopting a constructivist stance, the theoretical development emerged through the co-construction between the researcher and the participants. For this reason, addressing the researcher’s positionality was essential. The principal investigator (MVC) was a Chilean nurse and academic. During his early nursing training, he was introduced to research in occupational health—a field he further developed during his postgraduate studies, initially focusing on psychosocial risks and later specializing in work–family interaction. Regarding his clinical experience, he primarily worked in ICUs, including during the COVID-19 pandemic, where he directly experienced the phenomenon under study.

### 2.3. Recruitment

Participant recruitment was conducted in two institutions in Chile: one public hospital and one private hospital to represent both sectors of the Chilean health and labor systems. In Chile, a mixed healthcare system exists, comprising both public and private networks of care [28]. Both hospitals are high-complexity centers, anonymized as H1 and H2. Data collection began in H1 through purposive sampling and continued in H2 following an iterative process using constant comparative methods and guided by theoretical sampling. Data collection began upon receiving approval from the scientific ethics committees and continued throughout the year 2024.

Regarding the inclusion criteria, the study included nursing professionals working in Intensive Care Units, as well as nurse administrators from Hospitals H1 and H2 selected for this study, and adult family members of the nursing professionals who participated in the research. Nurses who were on medical leave or absent from their workplace for any reason at the time of data collection were excluded from the study.

### 2.4. Data Collection

Three data collection methods were employed: non-participant observation, in-depth interviews, and document analysis, following constructivist grounded theory strategies [26,29,30]. The central question guiding data collection was as follows: How does the work–family interaction process unfold among ICU nursing professionals? Initially, non-participant observations were conducted. The format used for field notes is presented in (Supplementary File S2). This method provided valuable information regarding the physical–social characteristics of the environment, individual and collective actions, and the consequences of social behaviors [31]. All information was recorded in the notebook of the principal investigator, then later digitized and analyzed.

In addition, the observations informed the in-depth interviews, which included clinical nurses, nurse administrators, and nurses’ adult family members. The guiding questions and the format used for these interviews are presented in (Supplementary File S2). The individual interviews were audio-recorded, each with an average duration of 53 min, and subsequently transcribed verbatim for analysis. None of the interviews required repetition, and the transcripts were not returned to participants, primarily because the experiences were analyzed iteratively, in accordance with grounded theory strategies.

Finally, existing documents relevant to the field were considered as data. These included international conventions [5,6], the Chilean Constitution [32], labor laws [33], health legislation [34], and ministerial guidelines for occupational risk management [35]. These documents helped identify the social, economic, historical, cultural, and situational contexts [26]. In this regard, the documents were used to analyze the institutional and legal framework of the phenomenon of interest, capturing both the macro and meso levels in which it is embedded.

### 2.5. Data Analysis

Data analysis was primarily based on constant comparison and multi-level coding [23]. The analytical process began with the initial data collection, during which an initial coding phase was conducted, followed by focused coding and categorization, culminating in theory construction. Throughout this process, memo writing and the constant comparative method were employed consistently. The analysis was managed and conducted using ATLAS.ti version 25 (ATLAS.ti GmbH, Berlin, Germany).

Given the nature of grounded theory, the data were subjected to continuous interrogation during analysis. Interview transcripts were coded line by line, documents were coded word by word, and observational data were coded incident by incident [36]. Subsequently, focused coding was carried out, elevating the analysis to a higher level of abstraction by selecting codes with greater analytical power or generating new codes. Abduction—i.e., explanatory reasoning—was employed for the theorization—that is, the process of theoretical construction. The analysis began with concepts, followed by their relationships, leading to the development of a theory that explained the phenomenon underlying the data [37].

The core category, “the process of reconciling work and personal roles” was conceptualized as the central mediating category between the occupational and personal spheres of the worker. Pattern repetition was considered the saturation point for this study. This saturation had a theoretical purpose; therefore, data saturation was considered to be achieved when no new insights emerged within the main categories of the developing theory. Supplementary Material File S3 presents the coding process used for the development of the core category.

### 2.6. Rigor and Trustworthiness

This study adhered to the 13 criteria of rigor in constructivist grounded theory [38]. The fulfillment of each criterion is detailed below: 1. The principal investigator maintained methodological self-awareness, explicitly documented through memo writing. Furthermore, all methodological decisions taken during the study were recorded and are substantiated within this article. 2. The research process was guided by the recommendations of the classical literature in the field, and the project was supervised by two experienced researchers, one of them an experienced grounded theorist (NF and LA). The supervision sessions allowed for the discussion of methodological decisions and the consensus-based interpretation of the data. 3. An open, critical, and analytical stance was adopted toward the existing literature, as demonstrated in the review of previous studies cited in this manuscript. 4. Rich and relevant data were collected, focusing on the main actors involved in the work–family interactions among ICU nurses. 5. Methodological decisions were transparently outlined throughout the study. 6. Data collection followed an iterative process, with the researchers moving continuously between data and analysis. 7. Ambiguity was tolerated as the understanding of the empirical world deepened, and this process was recorded through memo writing. 8. Progressively refined questions were developed, as exemplified in Supplementary Material File S2. 9. Theoretical explanations were sought within the data, as evidenced in the results and discussion sections, where explicit reference is made to the consulted theories. 10. Sufficient and varied data were collected to allow for meaningful comparisons and to support emerging categories. 11. Constant interrogation of the categories was conducted, allowing for the justification of their properties and theoretical scope. 12. Codes, categories, and theoretical frameworks were treated as provisional. Similarly, the emergent theory was regarded by the authors as substantive, explaining the data within a specific sociocultural, historical, and political context. 13. Following the analysis, the findings were contrasted with the existing literature.

Grounded theory practices such as multi-level coding, constant comparison, and memo writing were employed. Credibility was ensured through the analysis of participants’ experiences, which guided the theorization process. The use of gerunds and in vivo codes facilitated a focus on processes and helped uncover meanings that reflected the lived realities and experiences of nurses, nurse administrators, and family members. The final criterion for concluding the grounded theory process was theoretical transferability [39], that is, ensuring that the developed concepts and theories had applicability beyond the specific context and situation in which they were initially identified. Accordingly, their applicability and feasibility were supported by including two institutions from different settings (public and private). With the level of abstraction achieved, the findings transcended particularities (H1), allowing for their transfer to another context (H2).

### 2.7. Ethical Considerations

The project was reviewed and approved by the Scientific Ethics Committee of the Faculty of Nursing at Universidad Andrés Bello (L4/CECENF/01-2024), accredited by the Chilean government. Additionally, expedited reviews were conducted by the ethics committees of the participating institutions. Approval was granted by the Scientific Ethics Committee of the Western Metropolitan Health Service (10/2024) (public institution), and by the ethics committee of the private institution (162-11-24).

Informed consent was obtained from each participant, both orally and in writing, prior to their involvement. Confidentiality was maintained throughout the study; each participant was assigned a unique participation code.

## 3. Results and Discussion

To illustrate the connections between the empirical data and the theoretical literature, the results and discussion are presented jointly. This approach allows for a more explicit exposition of the abductive reasoning process [40].

The data analyzed in this study comprised 202 h of observation, 57 documents, and 51 in-depth interviews. Table 1 presents the demographic data of nurses and nurse administrators, while Table 2 provides the demographic data of the nurses’ family members.

**Figure 1:**
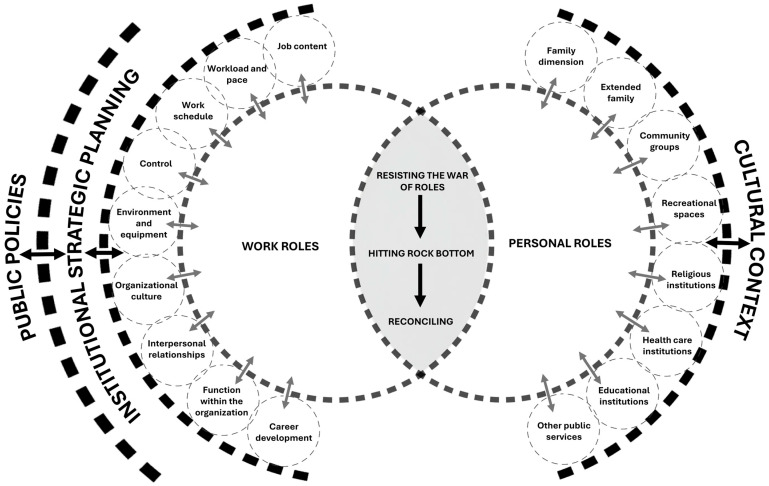
Process of reconciling work and personal roles.

“Reconciliation of work and personal roles” constitutes the core category that explains the interaction process between the professional and personal spheres of ICU nurses. Grounded theory aims to develop a theory grounded in a social process [23,26,41,42]. A social process is a type of core category characterized by a processual nature, meaning that it involves two or more clearly emerging stages [43]. In this context, “reconciliation of work and personal roles” is understood as a process composed of three stages: “resisting the war of roles”, “hitting rock bottom”, and “reconciling”; these stages were identified through the process of theorization. Through the reconciliation process, nurses’ work and personal roles, when in conflict, tend to move toward harmonization (see Figure 1).

**Figure 2:**
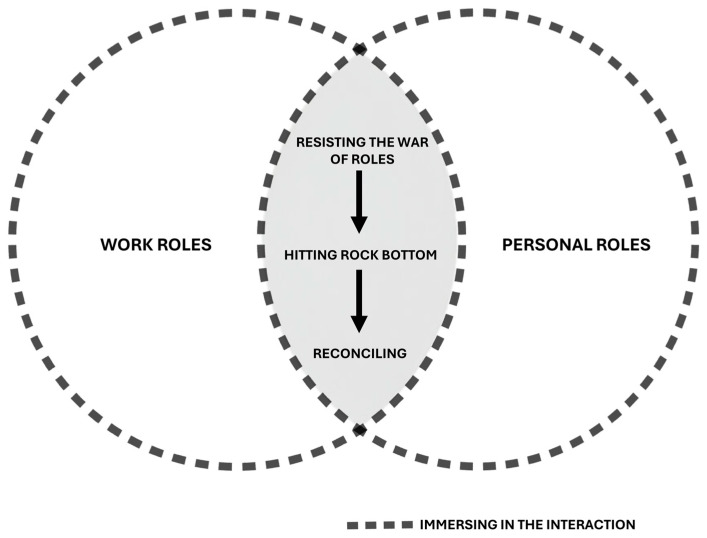
Core categories of the process of reconciling work and personal roles.

The core category is situated within a complex relationship between work and personal roles. Due to its inherent nature, a comprehensive exploration of this category cannot be fully addressed within a single manuscript. Therefore, this article presents the central process where the interaction between both roles takes place (see Figure 2).

### 3.1. Immersing in the Interaction

There are various means through which the interaction—“immersing in the interaction”—is facilitated, some of which are clearly identifiable, while others may be implicit or less visible. This is illustrated in the pictographic representation (Figure 2) by dashed lines connecting different sources of interaction, as an attempt to make tangible a flow of communication that is not always perceptible in practice—a metaphor adapted from the work–family border theory [44].

**Figure 3:**
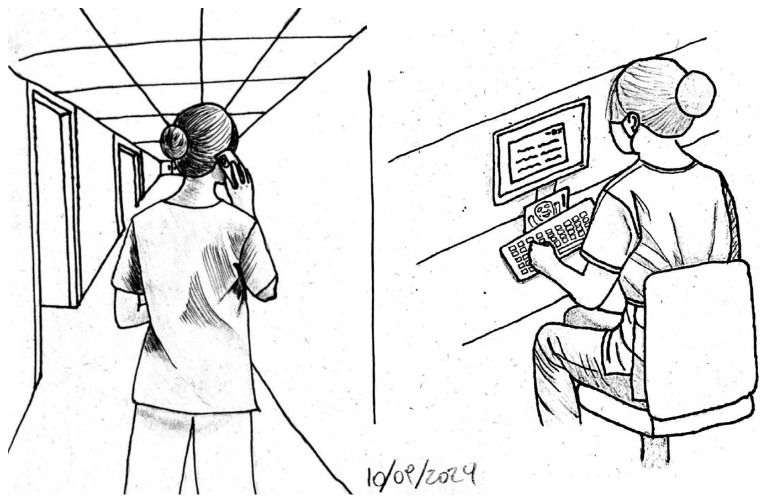
Field notes from observations.

The nature of this interaction can be classified into direct and indirect means. Direct means include situations in which the worker exhibits observable behaviors that reflect their experienced interactions, such as arriving home visibly discouraged after a physically and emotionally exhausting workday. Likewise, interaction may occur remotely, through phone calls, video calls, or text messages. Conversely, indirect interaction happens through self-reflection on concerns, responsibilities, or memories. The following section presents selected examples from observations (Figure 3) and interviews.

*“My female colleagues are constantly on video calls, coordinating lunch, coordinating everything. Like tomorrow—my colleague here—her daughter has to go do sports, and the day after, her son has to go somewhere else. And she’s here, doing her work while coordinating everything with her children”*.

(Interview, female nurse 29, ICUa, H2)

Thus, interaction may manifest in various forms—ranging from overt expressions such as a video call to more implicit ones, such as a reflective process concerning worries originating from sources of interaction, such as the family.

### 3.2. Understanding Roles

Initially, this study was structured around the idea of a bidimensional interaction between work and family—an approach that has predominated in the field [21]. Within this framework, numerous and diverse metaphorical proposals have been employed by the scientific community in an attempt to capture the complexity of the phenomenon. Some notable examples include work–family conflict [45], work/family border theory [44], work/life balance [46], work–home interference [47], work–home interaction [48], work–family facilitation [49], and work–life integration or balance [50]. However, the data revealed a complex network of interaction that transcended this traditional framework, compelling a conceptual shift from the notion of “family” to a broader understanding rooted in the concept of “roles”.

Adapting Biddle’s definition, roles are understood as the set of behaviors associated with the context under analysis. When the context refers to the work sphere, the term work roles will be used, and when it refers to the personal sphere, it will be referred to as personal roles [51].

#### 3.2.1. Work Roles

The behaviors of ICU nurses are shaped by various sources of interaction within the workplace, which are influenced by institutional strategic planning—and, in turn, by both national and international public policies [52]. These sources of interaction may exert a negative synergistic effect; that is, when one source enters into conflict, it can compromise others, thereby amplifying the negative impact on the worker. Conversely, when negative and positive sources of interaction coexist, the latter can mitigate the negative effects [53].

Work roles are determined by the sources of interaction present within the ICU context. These include (Figure 1) the following: job content; workload and pace; work schedule; control; environment and equipment; organizational culture; interpersonal relationships; function within the organization; and career development.

#### 3.2.2. Personal Roles

The behaviors of nurses in their personal sphere are shaped by various sources of interaction, primarily within their cultural context [54], where the family is understood as one social subsystem among many. Consequently, a comprehensive understanding of the family requires an analysis of its relationship with other social subunits, without which the phenomenon would be only partially understood.

In this regard, personal roles depend on the sources of interaction present in each individual, which include (Figure 1) the following: family dimensions; extended family; community groups; recreational spaces; religious institutions; healthcare institutions; educational institutions; and other public services.

### 3.3. The Process of Reconciling Work and Personal Roles: Resisting the War of Roles

The first stage in the process of reconciling work and personal roles is “resisting the war of roles”, defined as a chaotic process characterized by the constant flow of demands between the worker’s professional and personal roles. The use of the term “war” in the name of this stage serves as a metaphor to represent the tension and incompatibility between different roles. While the participants did not explicitly use the word “war”, their narratives conveyed a strong sense of conflict and struggle. Therefore, “war of roles” captures the chaotic and exhausting nature of this phenomenon, as experienced by the participants going through this stage.

*“But you go through a period when you’re more tired—it happened to me at the beginning, when I changed units. I would come home completely exhausted. I think it was everything at once, because there was also emotional fatigue due to the change, new people, and this unit is more challenging, it’s more difficult. I had never worked in the ICU before, and I was assigned to the ICU. During that time, I felt it was harder for me to reconcile work and family life because I was so tired”*.

(Interview, female nurse 28, ICUa, H2)

Each role has the capacity to interact internally with its own sources of interaction and externally with the sources of interaction associated with the other role. It is a complex process in which all of the workers’ available resources converge, with a clear tendency toward resistance in the face of conflict.

*“I think that when I first started working… I had a really hard time. I was constantly, um… leaving work feeling bad. I would punish myself for having done something wrong. I also carried it with me, feeling bad deep down, because I wasn’t doing things well, because I was told I wasn’t doing things well. So, yes, I think I definitely carried that with me”*.

(Interview, female nurse 12, ICUa, H2)

The specialized nature of ICUs, where the focus of care is on critically ill patients, requires an advanced level of technical skills and biomedical knowledge [55]. This demand is particularly significant for nurses who are encountering such patients for the first time—usually during the early stages of their professional careers—but it is also present among more experienced nurses who transition into Critical Care Units. This observation aligns with Benner’s theory: from novice to expert [56]. In this context, “resisting the war of roles” begins as soon as nurses enter a Critical Care Unit.

*“When you’re new, you’re attentive to everything your boss says, everything your colleagues say. And over time, of course, since you’re new, you’re hyperaware of everything—for example, even on your days off, you’re still thinking about everything that was discussed. Basically, you don’t disconnect from work. It’s typical to be contacted outside of your working hours, and you end up normalizing it — being spoken to about work matters outside your shift”*.

(Interview, male nurse 15, ICUa, H1)

However, interactions are not limited to dynamics perceived as harmful by workers, as illustrated in the four-dimensional model proposed by Geurts et al. [48]. In this model, negative strain experienced at work can hinder performance at home, and conversely, negative strain within the family can impede performance at work. At the same time, positive work experiences can facilitate functioning in the home sphere, and likewise, positive family experiences can enhance performance in the workplace.

*“We cannot avoid the fact that work affects personal life, and that personal life affects work—these are aspects that are completely interconnected. You will never be able to separate them; both influence and impact each other, for better or worse…”*.

(Interview, nurse administrator 1, ICUp, H1)

Regarding the positive influence of the nurse’s personal role on their professional role, it is reflected through life experiences and milestones that contribute to the acquisition of tools for better performance in the workplace. These include values acquired through family upbringing and marital relationships, such as loyalty, responsibility, commitment, honesty, kindness, and organizational skills. Additionally, experiences with institutions such as religious organizations are perceived as opportunities where nurses can develop communication and emotional support skills—even prior to receiving formal professional training.

Moreover, taking on a parental role—being a mother or father—enables nurses to cultivate a heightened sensitivity in caregiving, often perceived as more affectionate or compassionate care. Finally, experiencing critical life events, such as the illness of a loved one and undergoing the process of being a patient’s family member, allows nurses to develop deeper empathy and a greater understanding of the nuances of their professional responsibilities—key elements in professional development.

This type of positive influence aligns with findings from a qualitative study conducted with nurses in the United States, which described how personal experiences enriched their professional practice. For example, becoming a parent enhanced their ability to care for patients, while having close relationships with loved ones facing challenges improved their capacity to connect with and support patients with similar issues [57].

The following is a narrative from a nurse describing how their family has influenced their work:

*“I believe my father’s example—especially in his work and in his life—was that of a good man. That’s how I would define him: a good man. Good in the sense of being honest. And I think that example is present in my own work, particularly in terms of commitment, honesty, responsibility, and also kindness—not the kind of kindness that tries to please everyone, but the kind that, if there is a chance to do good, then you do it; if you can avoid harming others, then you don’t; to be fair. And regarding my family, my partner plays such an important role, especially because of her ability to reconcile different aspects of life and her strong sense of responsibility. I think the essence I already carried within me has become more organized with her—more structured, more disciplined. And my son… well, he is my driving force. Everything I do, I do think of him. I see him everywhere—I see him in my patients, for example. Before, I might have just been kind and respectful, but since my son was born, I feel I’ve become more affectionate. Not with everyone, but I am more affectionate in that sense, because I’ve learned to express a little more love”*.

(Interview, male nurse 25, ICUa, H2)

Regarding the positive influence of the work role on the family role, employment was highlighted as a means for nurses to develop financial autonomy, which is essential for securing housing, affording better education for their children, and accessing comforts such as owning a personal vehicle or traveling—ultimately contributing to the family’s overall well-being. These findings are consistent with those reported in a qualitative study of Turkish nurses [58], which emphasized the economic contributions of nursing work as a positive benefit in the lives of their families.

Moreover, in situations involving conflict within personal roles, work can function as an emotional buffer by providing distraction. It may also serve as a facilitator for problem-solving—for example, through flexible work schedules. Finally, being a nurse can be a source of pride and recognition among peers, extended family, and community groups, contributing to the worker’s sense of self-fulfillment.

*“For example, at the end of the year we organize the premature babies’ celebration… And seeing those little ones who are now big kids—healthy, intact, normal—and laughing like any other child, even though they once weighed, I don’t know, 600 grams… it truly feels like an achievement. For me, it brings a sense of pride, it makes me happy that our work is reflected in the fact that these children are there, playing with their parents and siblings. It feels good to share that with them. I get home, and it’s like I still have that smile on my face—like it’s been imprinted”*.

(Interview, male nurse 35, ICUp, H1)

These findings are in line with the Professional Quality of Life Model [59], which defines professional quality of life based on two components, one of which is compassion satisfaction—positive feelings about one’s ability to help others, as well as the pleasure derived from performing the job well. The concept emerged from the study of professionals exposed to traumatic situations and was developed under the premise that it is impossible to understand the negative aspects of caregiving without also acknowledging the positive ones. Additionally, the model recognizes other influential factors, such as the importance of protecting the caregiver’s reputation in the eyes of their employer [60].

Regarding the negative influence of the nurse’s personal role on their professional role, a key issue identified was the overlap of personal responsibilities, which makes it difficult to comply with strict work schedules—for instance, morning shifts coinciding with children’s school drop-off times or the need to leave a shift due to unforeseen family circumstances. Additionally, beginning the workday already fatigued as a result of reproductive labor was noted as a challenge.

Furthermore, interpersonal conflicts with close family members or significant others were reported to affect nurses’ communication with colleagues, patients, and their families—manifesting in changes in attitude, mood, and irritability. Lastly, persistent concerns about personal role responsibilities were shown to negatively impact the worker’s concentration.

*Interviewer MVC [Author]: Could you describe a situation in which you believe your personal life influenced your work? Nurse 28: For example, when I separated from my ex-partner—sometimes you just can’t separate things. I remember that shift was awful; I reacted terribly and was very distracted. In the end, I spoke with my supervisor, and I had to leave because I kind of had a breakdown*.

(Interview, female nurse 28, ICUa, H2)

Another participant mentioned the following:

*“It’s horrible—there’s no time, no time at all. We don’t have time. The exhaustion… and that’s one of the reasons why I still don’t have children, because I don’t know how I would divide my time”*.

(Interview, female nurse 33, ICUp, H1)

Another negative influence of work role interaction sources on personal roles can be the difficulties in forming and projecting a family. In addition, insufficient time for fulfilling personal roles was noted, particularly due to failure to comply with the scheduled end of the workday.

Other aspects included changes in behavior at home, such as mood, interactions with family members, pace of life, and fatigue, which in turn affected the ability to perform reproductive labor, participate in family- and school-related activities, and engage in leisure time. Finally, physiological functions such as breastfeeding could be compromised due to a lack of adequate time and physical conditions to support them.

Our findings are consistent with those reported in other studies. For instance, Skinner et al. [61], in their qualitative study of healthcare professionals, reported that some participants wished to have children but felt unable to do so in the near future due to their working conditions. This phenomenon was later conceptualized by Cottingham et al. [57], who introduced the term “anticipatory spillover” to describe the anticipation of future work–family conflict among young nurses.

On the other hand, when exploring how the family knew that the nurse had a difficult day at work, one participant stated the following:

*“Because she comes home tired, I see it—her face, the dark circles under her eyes. She doesn’t want to do anything. She comes in, throws herself on the bed, takes off her clothes and leaves them on the floor… she’s unmotivated. You can tell—she doesn’t want to talk, doesn’t want to engage in conversation. She just wants to sit and watch TV or her shows, with no interaction. That’s when I say, ‘Alright, it was a rough shift.’ Then I ask her how it went, and that’s when she tells me”*.

(Interview, nurse’s family member 4)

This first stage, “resisting the war of roles”, converges at a point where the conflict becomes unsustainable, perceived as “hitting rock bottom”.

### 3.4. The Process of Reconciling Work and Personal Roles: Hitting Rock Bottom

The second stage in the process of reconciling work and personal roles is “hitting rock bottom”, defined as a process in which the worker becomes aware of the need to initiate change in the management of their roles. This represents a critical turning point that prompts the worker to modify their work and personal routines by drawing on the available resources from their sources of interaction, thus enabling progression toward the next stage: “reconciling”.

*“I had a crying episode, right there at the table while having lunch with my family, with a family member sitting next to me looking at me as if to say, ‘what’s wrong with you?’ And when I saw her, I thought: no, I can’t keep wasting time thinking about what is happening at work and wasting this valuable and limited time I have with my family. I feel that was the trigger, so to speak. It cannot be that I stop enjoying the moment because of what work might cause me. At that point, it became clear that I had to do something. In fact, that same day I started looking and researching which psychologist I could go to…”*.

(Interview, nurse administrator 7, ICUp, H1)

Hitting rock bottom can result from a range of situations, from self-reflection to the loss of a loved one. Self-reflection arises when individuals consciously analyze their emotions and experiences—for example, becoming aware of the aging of family members and feeling the need to prioritize time with them, or facing emotional crises stemming from work overload. Marital conflicts, ranging from arguments to more complex situations such as divorce, align with the findings of a recent review in the field. Kelemen et al. describe the various intersections between work and divorce, including divorce as a consequence of marital conflict [62].

In the workplace, exposure to critical incidents and unethical institutional practices can challenge professional values, leading workers to question their continued employment or to feel devalued after being dismissed.

*“I believe that, driven by anxiety, I hit rock bottom at some point. I would suddenly find myself sitting and thinking, ‘Why do I feel so anxious if I have nothing tomorrow?’ For instance, tomorrow is Saturday, there’s nothing scheduled. And yet I would feel that weight on my chest, palpitations, and everything. I would ask myself, ‘What is going on? I’m at home, doing nothing, just quietly watching a movie.’ It was like—I can’t live like this anymore. So I went to a psychiatrist, a psychologist, the whole thing. They put me on medication, but nothing changed. Then I realized that when I started working, I had stopped exercising. Exercise used to be a habit for me, a family habit. So I had abandoned it, and it actually coincided a bit with the onset of these symptoms… That’s when I said, ‘Alright, I need to make a change. I want to do something.’ Honestly, the pillar for me is my family”*.

(Interview, female nurse 4, ICUa, H1)

Similarly, another related situation involves health, as occupational burnout can lead to illness that forces individuals to reassess their lifestyle. Finally, the loss of loved ones often triggers feelings of guilt for not having spent enough time with them—or, in the case of patients, particularly when on their deathbed, some express that excessive work prevented them from enjoying life.

### 3.5. The Process of Reconciling Work and Personal Roles: Reconciling

The third stage in the process is “reconciling”, defined as a process in which the worker’s sources of reconciliation converge synergistically. Although sources of potential conflict may still be present, what distinguishes this stage is the acquisition of tools that enable the worker to effectively reconcile the interaction between work and personal roles.

*“The difference lies in the experience you have in the job—it allows you, in a way, to discern what is truly important from what might not be so important, to recognize what holds value and what is more routine. In that sense, of course, each person has their own perspective, but ultimately, there are things that only time and experience enable you to see clearly and to take a certain distance—to decide what deserves your attention, your time, and your energy. I mean, where you choose to place emphasis”*.

(Interview, female nurse 10, ICUa, H2)

Nurses who find themselves in this stage are often experienced professionals who have developed resilience through a personal and experiential process, which therefore varies from one individual to another. This process follows an intuitive path of maturation and the development of emotional intelligence. This notion is supported by a literature review on resilience and emotional intelligence in nurses [63], which describes the existence of both internal and external protective factors for the development of resilience. Among the internal factors is emotional intelligence—that is, the set of skills that helps individuals understand themselves and others; be aware of their own emotions and those of others; build interpersonal relationships; find flexible, realistic, and effective solutions to problematic situations; and adapt to and cope with irresolvable challenges.

On the other hand, external factors include social support, professional experience, and peer support—resources that, within our proposal, stem from the worker’s personal and work-related sources of interaction.

These findings are consistent with studies conducted in ICUs, which indicate that more experienced professionals tend to report higher levels of compassion satisfaction [64,65,66]. This suggests that, over time, nurses develop resilience to workplace stressors [67].

*“One learns to prioritize in these units because you’re always walking on the edge, on the edge, on… on the brink of the abyss—between whether they live or die. As nurses who work in critical care, we have a significant dose of resilience; we recover from one case after another, from one after another…”*.

(Interview, nurse administrator 2, ICUa, H2)

*“But there comes a moment when you realize that it does take a toll—that it is not necessary to be empathetic to the point of actually feeling the other person’s emotions. You have to be empathetic, yes, but there must be a boundary. And that boundary… you’re not born knowing where it is. That’s where professionals who are trained in these matters can help us. I came to understand this after having worked for about ten years. I wish I had discovered it earlier, because after that, it was as if my mind said, ‘You’ve discovered that psychologists exist…’”*.

(Interview, male nurse 32, ICUa, H1)

In conclusion, the process of reconciling work and personal roles is complex and encompasses multiple elements. Our proposal highlights the importance of studying the various sources of interaction associated with each role from an integrated perspective—a crucial approach for advancing the understanding of the phenomenon.

## 4. Strengths and Limitations

Among the strengths of this study is its comprehensive approach to the phenomenon, offering a detailed description of both the work-related and personal sources of interaction among ICU nurses. In doing so, it addresses the theoretical gaps identified by the scientific community [21]. Likewise, the present study offers a process-oriented approach to the phenomenon, establishing a discussion focus that has been scarcely addressed to date, while considering the ethical challenges it entails [22]. The proposed theory underscores the complexity of the phenomenon, providing compelling evidence that it encompasses far more than a simple work–family interaction. The sources of interaction described in the present study, both work-related and personal, provide valuable information for decision-makers and will also complement management models in the field.

This study is contextual and hence the primary limitation lies in the sociocultural context, which may restrict the transferability of the findings, as the studied reality pertains to the Chilean sociocultural demands and extended 12 h work shifts. Additionally, data collection took place in 2024, a post-COVID-19 pandemic period during which some narratives still reflected the emotional sensitivity associated with pandemic-related experiences. On the other hand, it is important to mention that the present study reports on the core process of the theory; work roles and personal roles will be addressed in separate manuscripts, as the complexity of the phenomenon makes it impossible to explain its development in a single publication.

## 5. Conclusions

This study developed a theory that explained the process of reconciling work and personal roles among ICU nurses in Chile from the perspective of the nurses themselves, their families, and nursing supervisors. Role reconciliation emerged as the basic social process that explained the interaction between nurses’ work and personal roles. This process consisted of three stages: resisting the war of roles, hitting rock bottom, and reconciling.

This study highlights the importance of considering the multiple sources of interaction within each role; without this consideration, the analysis would remain incomplete. In doing so, it responds to a need identified by the scientific community and offers new insights into the understanding of the phenomenon—insights that will be crucial for effective intervention. Future proposals should provide evidence regarding the degree of transferability of these findings, especially when accounting for cultural particularities.

## Figures and Tables

**Table 1:** Demographic data of clinical nurses (*n* = 38) and nurse administrators (*n* = 8).

	Clinical Nurses (*n* = 38)	Nurse Administrators (*n* = 8)
*Gender*		
Male	10	0
Female	28	8
*Age*		
25–30	4	0
31–40	24	3
41–50	8	2
51–65	2	3
Unit *		
Intensive Care Units, adult (ICUa)	32	6
Intensive Care Units, pediatric/neonatal (ICUp)	6	2
*Children/Dependents Under Their Care*		
No	22	4
Yes	16	4
*Work Experience (years)*		
1–10	13	1
11–20	22	4
21–30	2	2
31–50	1	1
*Experience in Current Position (years)*		
1–10	31	1
11–20	6	7
21–30	1	0

* ICUa includes adult Critical Care Units, both general and specialized (medical, surgical, burn, neurocritical, and coronary units); ICUp includes Critical Care Units for pediatric and neonatal patients.

**Table 2:** Demographic data of nurses’ family members (*n* = 5).

Demographic Data	*n*
*Gender*	
Male	3
Female	2
*Age*	
18–30	1
31–40	3
41–50	1
*Occupation*	
Worker	4
Student	1

## Data Availability

For confidentiality purposes, the data are in the possession of the author (MVC).

## Supplementary Materials

The following supporting information can be downloaded at: https://www.mdpi.com/article/10.3390/healthcare13101206/s1, File S1: COREQ checklist; File S2: Data collection form; File S3: Coding process. Reference [68] is cited in Supplementary Materials.

## Abbreviations

The following abbreviations are used in this manuscript:

ICU	Intensive Care Unit
