# Supplementary material for: Reconciliation of Work and Personal Roles Among Critical Care Nurses: Constructivist Grounded Theory Research
Source: Healthcare (Basel). 2025 May 21;13(10):1206. doi: 10.3390/healthcare13101206 (PMC12111176; doi:10.3390/healthcare13101206)
Supplement: Supplementary file 1 [file healthcare-13-01206-s001.zip › S2.pdf]

## DATA COLLECTION FORM

**Non-Participant Observation**

Date: ____ / ____ /202__ Time: _________

Location: ______________________________________________________________________

| **Observation Specification** | **Description** |
|---|---|
| Individual and collective actions. | |
| Detailed notes, including anecdotes and observations. | |
| Emphasis on significant processes occurring in the environment. | |
| Consideration of what participants define as interesting and/or problematic. | |
| Attention to participants' language use. | |
| Contextualization of actors and actions within scenes and settings. | |

**Work charts: schemes, diagrams, and maps**

## IN-DEPTH INTERVIEWS

**Date: ____ / ____ /202__** **Participation Code: ______________________**

| **Sociodemographic Aspects** | |
|---|---|
| Would you like to be referred to by a pseudonym? | |
| What gender do you identify with? | |
| What is your date of birth? | |
| Do you have children or family members under your care? | |
| Considering all the jobs you have had throughout your life; how long have you been working in total? | |
| How long have you been working in your current unit (job)? | |

| **GUIDING QUESTIONS** | | |
|---|---|---|
| **Nurses** | **Family Members of Nurses** | **Nurse Administrators** |
| -How is your routine on a day when you have to go to work?<br>-What are your thoughts on the relationship between work and family life?<br>-Tell me about a situation where you believe your work has influenced your family life.<br>-Tell me about a situation where you believe your family life has influenced your work. | -How is the routine on a day when your relative has to go to work?<br>-What are your thoughts on the relationship between work and family life?<br>-Tell me about a situation where you believe your relative's work has influenced family life.<br>-Tell me about a situation where you believe family life has influenced your relative's work. | -What are your thoughts on the relationship between work and family life of nursing staff?<br>-Tell me about a situation where you believe the work of nurses has influenced their family life.<br>-Tell me about a situation where you believe the family life of nurses has influenced their work. |
| *Notes*<br>______________________________<br>______________________________<br>______________________________<br>______________________________<br>______________________________<br>______________________________<br>______________________________<br>______________________________<br>______________________________<br>______________________________ | | |
