# Supplementary material for: Reconciliation of Work and Personal Roles Among Critical Care Nurses: Constructivist Grounded Theory Research
Source: Healthcare (Basel). 2025 May 21;13(10):1206. doi: 10.3390/healthcare13101206 (PMC12111176; doi:10.3390/healthcare13101206)
Supplement: Supplementary file 1 [file healthcare-13-01206-s001.zip › S3.pdf]

# CODING PROCESS FOR THE DEVELOPMENT OF "WORK-LIFE ROLE RECONCILIATION"

<table>
<tr><th>Data</th><th>Initial codes (n=68)</th><th>Theoretical links</th><th>Category and focused codes (n=13)</th></tr>
<tr><td>"It is necessary for the management of work-life balance to be capable of considering the multiple vital roles..."<br>(Document, Chilean Institute of Public Health, 2022, p. 9)</td><td rowspan="4">Implications of Work-Family Interaction</td><td rowspan="4">What are the characteristics of the interaction?<br>What are the sources of interaction?<br>Why do some individuals perceive a reconciliation between roles while others do not?<br>Is it merely a positive/negative and bidirectional interaction, as proposed by Geurts et al. (2005)?</td><td rowspan="4">Core Category: Reconciliation of Work and Personal Roles*<br><br>Stage 1: Resisting the Role War<br>-Onset of conflict<br>-Positive influence of work roles<br>-Negative influence of work roles<br>-Positive influence of personal roles<br>-Negative influence of personal roles<br><br>Stage 2: Hitting Rock Bottom<br>-Self-reflection<br>-Marital conflicts<br>-Exposure to critical events and unethical institutional practices<br>-Health<br>-Loss<br><br>Stage 3: Reconciling<br>-Developing resilience<br>-Seeking reconciliation<br>-Implementing changes in daily routines<br><br>(*) Understanding the core category required conceptualizations of the work and personal roles of nurses working in critical care units.</td></tr>
<tr><td>"We cannot avoid the fact that work affects personal life, and that personal life affects work — these are aspects that are completely interconnected. You will never be able to separate them; both influence and impact each other, for better or worse..." (Interview, Nurse administrators 1, ICUp, H1).</td></tr>
<tr><td>"Someone very close to me passed away. While reflecting on it, I realized—wow! I hadn’t seen them in a long time. And why? Because I had been spending so much time working… life was slipping away from me. That’s when I started to think: what do I really want from my life?" (Interview, Female nurse 9, ICUa, H2).</td></tr>
<tr><td>"I have engaged in many activities to become the nurse who is speaking to you now—many things. It has been a process of construction. Obviously, I am not the same person I was years ago; I have been gradually building myself in order to take care of myself and my family" (Interview, Nurse administrators 5, ICUa, H2).</td></tr>
</table>
